# 3D-electroanatomical mapping of the left atrium and catheter-based pulmonary vein isolation in pigs: A practical guide

**DOI:** 10.3389/fcvm.2023.1139364

**Published:** 2023-03-09

**Authors:** Julie Norup Hertel, Kezia Jerltorp, Malthe Emil Høtbjerg Hansen, Jonas L. Isaksen, Stefan Michael Sattler, Benedikt Linz, Sevasti-Maria Chaldoupi, Thomas Jespersen, Arnela Saljic, Uffe Gang, Martin Manninger, Dominik Linz

**Affiliations:** ^1^Department of Biomedical Sciences, Faculty of Health and Medical Sciences, University of Copenhagen, Copenhagen, Denmark; ^2^Department of Cardiology, Gentofte University Hospital, Hellerup, Denmark; ^3^Emergency Department, Frederiksberg and Bispebjerg Hospital, Copenhagen, Denmark; ^4^Department of Cardiology, Cardiovascular Research Institute Maastricht (CARIM), Maastricht University Medical Centre, Maastricht, Netherlands; ^5^West German Heart and Vascular Center, Institute of Pharmacology, University Duisburg-Essen, Essen, Germany; ^6^Department of Cardiology, Zealand University Hospital Roskilde, Roskilde, Denmark; ^7^Division of Cardiology, Department of Internal Medicine, Medical University of Graz, Graz, Austria; ^8^Centre for Heart Rhythm Disorders, Royal Adelaide Hospital, University of Adelaide, Adelaide, SA, Australia

**Keywords:** pulmonary vein ablation/isolation, pig, transseptal puncture, multielectrode mapping, radiofrequency ablation

## Abstract

**Aim:**

To propose a standardized workflow for 3D-electroanatomical mapping guided pulmonary vein isolation in pigs.

**Materials and methods:**

Danish female landrace pigs were anaesthetized. Ultrasound-guided puncture of both femoral veins was performed and arterial access for blood pressure measurement established. Fluoroscopy- and intracardiac ultrasound-guided passage of the patent foramen ovale or transseptal puncture was performed. Then, 3D-electroanatomical mapping of the left atrium was conducted using a high-density mapping catheter. After mapping all pulmonary veins, an irrigated radiofrequency ablation catheter was used to perform ostial ablation to achieve electrical pulmonary vein isolation. Entrance- and exit-block were confirmed and re-assessed after a 20-min waiting period. Lastly, animals were sacrificed to perform left atrial anatomical gross examination.

**Results:**

We present data from 11 consecutive pigs undergoing pulmonary vein isolation. Passage of the fossa ovalis or transseptal puncture was uneventful and successful in all animals. Within the inferior pulmonary trunk 2–4 individual veins as well as 1–2 additional left and right pulmonary veins could be cannulated. Electrical isolation by point-by-point ablation of all targeted veins was successful. However, pitfalls including phrenic nerve capture during ablation, ventricular arrhythmias during antral isolation close to the mitral valve annulus and difficulties in accessing right pulmonary veins were encountered.

**Conclusion:**

Fluoroscopy- and intracardiac ultrasound-guided transseptal puncture, high-density electroanatomical mapping of all pulmonary veins and complete electrical pulmonary vein isolation can be achieved reproducibly and safely in pigs when using current technologies and a step-by-step approach.

## Introduction

Pulmonary vein isolation (PVI) is the cornerstone of invasive atrial fibrillation (AF) therapy (1). Multiple animal models have been utilized for pre-clinical development and refinement of various novel innovative catheter-based ablation strategies (2–5). Although the pig has been widely used for catheter development as well as for training purposes due to a postulated large similarity between the cardiac anatomy of pigs and humans, a detailed characterization of the left atrial anatomy using state of the art clinically available technologies is lacking.

A systematic practical guide of catheter based transseptal puncture and 3D-electroanatomical mapping and ablation strategies will support the principle within the 3Rs (replacement, reduction, refinement) framework for performing more humane animal research (6). Discussing the selection of the best technologies and communicating challenges during the implementation of a new animal model will help to achieve robust and reproducible results and reduce procedure-related complications, which serves both aspects “reduction” and “refinement” of the 3R principle.

Here, we present a practical guide for a standardized workflow for catheter-based electroanatomical mapping and pulmonary vein isolation in pigs considering the species-specific anatomy and catheter-related procedural/technical challenges available in the current literature.

## Materials and methods

The experimental protocol was approved by the Regional Animal Welfare Inspectorate (Miljø- og Fødevareministeriet: license #2018-15-0201-01608) and conforms with the guidelines from Directive 2010/63/EU of the European Parliament on the protection of laboratory animals and conforms with the Guide for the Care and Use of Laboratory Animals published by the US National Institute of Health (NIH publication no. 85–23, revised 1996).

### Anaesthesia protocol

All pigs were housed with *ad libitum* drinking water, hay, and regular feed (1.4 L/50 kg, Brogaarden) twice daily. The pigs were fasted and pre-medicated with a Zoletile-mixture intramuscularly (0.14 mL/kg; Zolazepam+Tilatemin 250 mg, Virbach, Kolding, Denmark; 6.5 mL Xylazine 20 mg/mL, CP-Pharma, Burgdorf, Germany; 1.25 mL Ketamine 100 mg/mL, Ricther Pharma AG, Wels, Austria; 2.5 mL Butorphanol 10 mg/mL Biovet Aps, Fredensborg, Denmark; and 2 mL Methadone 10 mg/mL Eurovet Animal Health B.V., AE Bladel, Netherlands), and brought to the operation theater, put into a supine position on the operation table and intubated. Anesthesia was induced with an intravenous bolus of α-Chloralose 4.2% (8 mg/kg, Sigma-Aldrich Chemie GmbH, Steinheim, Germany), maintained by a continuous infusion of α-Chloralose 4.2% (6.5 mg/kg/h) and Fentanyl (5 μg/kg/h, Hameln Pharma GmbH, Hameln, Germany), allowing spontaneous respiration. The anesthesia was supplemented with Zoletile-mixture intramuscularly as needed. Oxygen saturation was recorded with tail oximetry, temperature was monitored with a rectal thermometer, and arterial blood-gas was monitored regularly from a catheter (Seldinger arterial catheter, 22-gauge, Arrow International, Reading, United States) in the femoral artery. A urinary catheter was inserted into the urethral opening and advanced into the urinary bladder.

### Vascular access

Local anesthesia (Xylocaine 10 mg/mL, Aspen Pharma, Dublin, Ireland) was administered subcutaneously in the midline of the neck (5 mL) and in each groin (5 mL).

Neck access by cutdown: A ventral midline incision of 5–7 cm was performed to access the left carotid artery and both internal jugular veins, and three 8F sheaths (Avanti+ Introducers, Cordis Corporation, Florida, United States) were placed.

**Figure 1:**
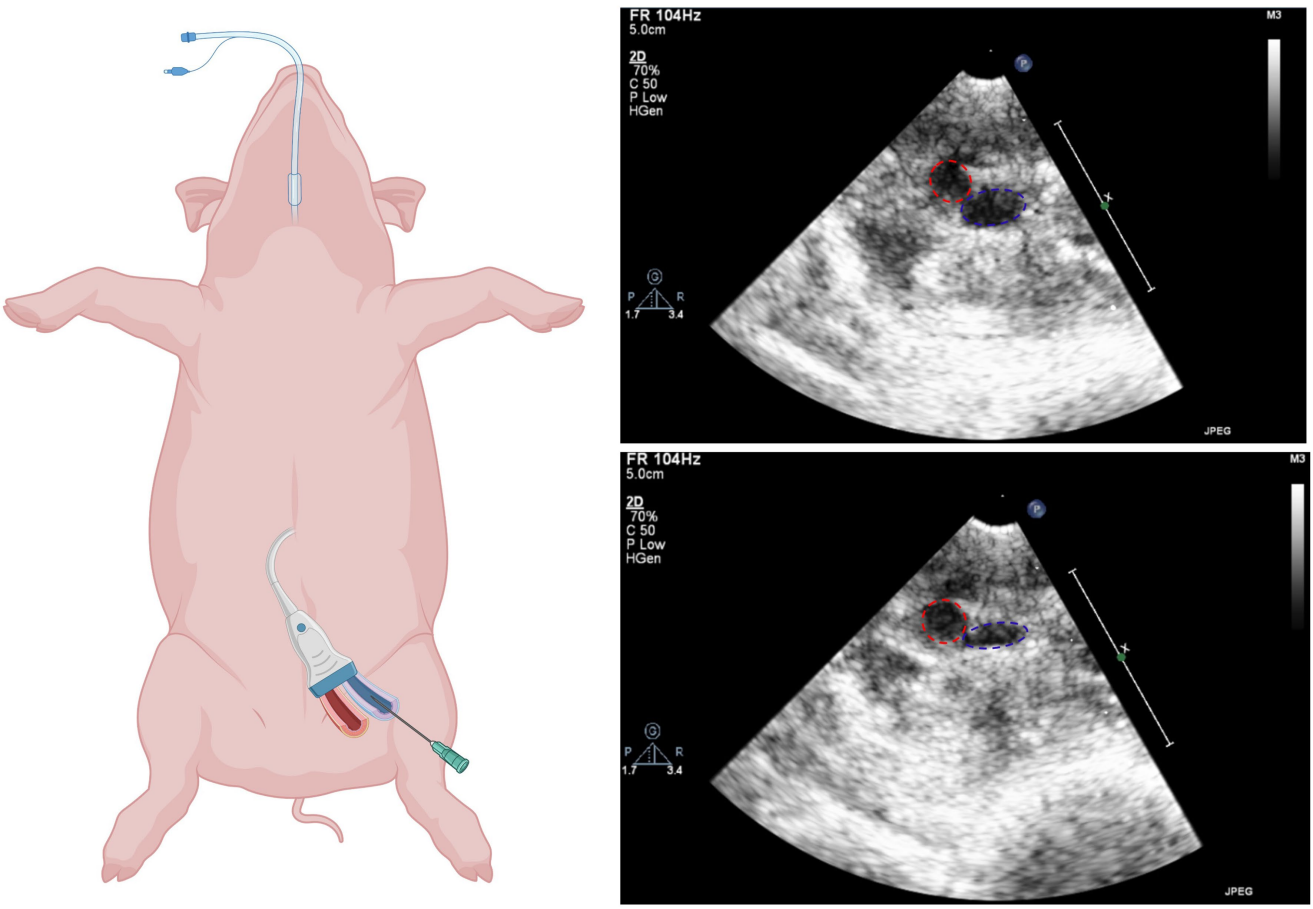
Ultrasound guided femoral access. Left: Schematic illustration of vascular access (biorender.com). Right: Ultrasound images of femoral artery (red) and femoral vein (blue) before (top) and after (bottom) compression.

Ultrasound-guided groin access: The femoral artery and veins were located using ultrasound and tested for compressibility (veins completely compressible, arteries remain spheric). An angiographic needle (18 G, 70 mm, Cordis Cashel, County Tipperary, Ireland) was used to access the veins and to place an 8F sheath *via* a guidewire (Seldinger technique; Figure 1).

Following vascular access, a bolus of 10,000 IE unfractionated heparin (Leo Pharma A/S, Lysaker, Norway) was administered, and a continuous infusion of 5,000 IE/h started.

Two decapolar diagnostic catheters (Inquiry™, 6F, Abbott) were advanced through the jugular veins into the coronary sinus and high right atrium under fluoroscopic guidance. A pressure transducer (Micro-tip Catheter Transducer, 5\u00B0F Milar Inc., Houston, United States) was placed in the left ventricle *via* the carotid artery.

### Transseptal puncture

**Figure 2:**
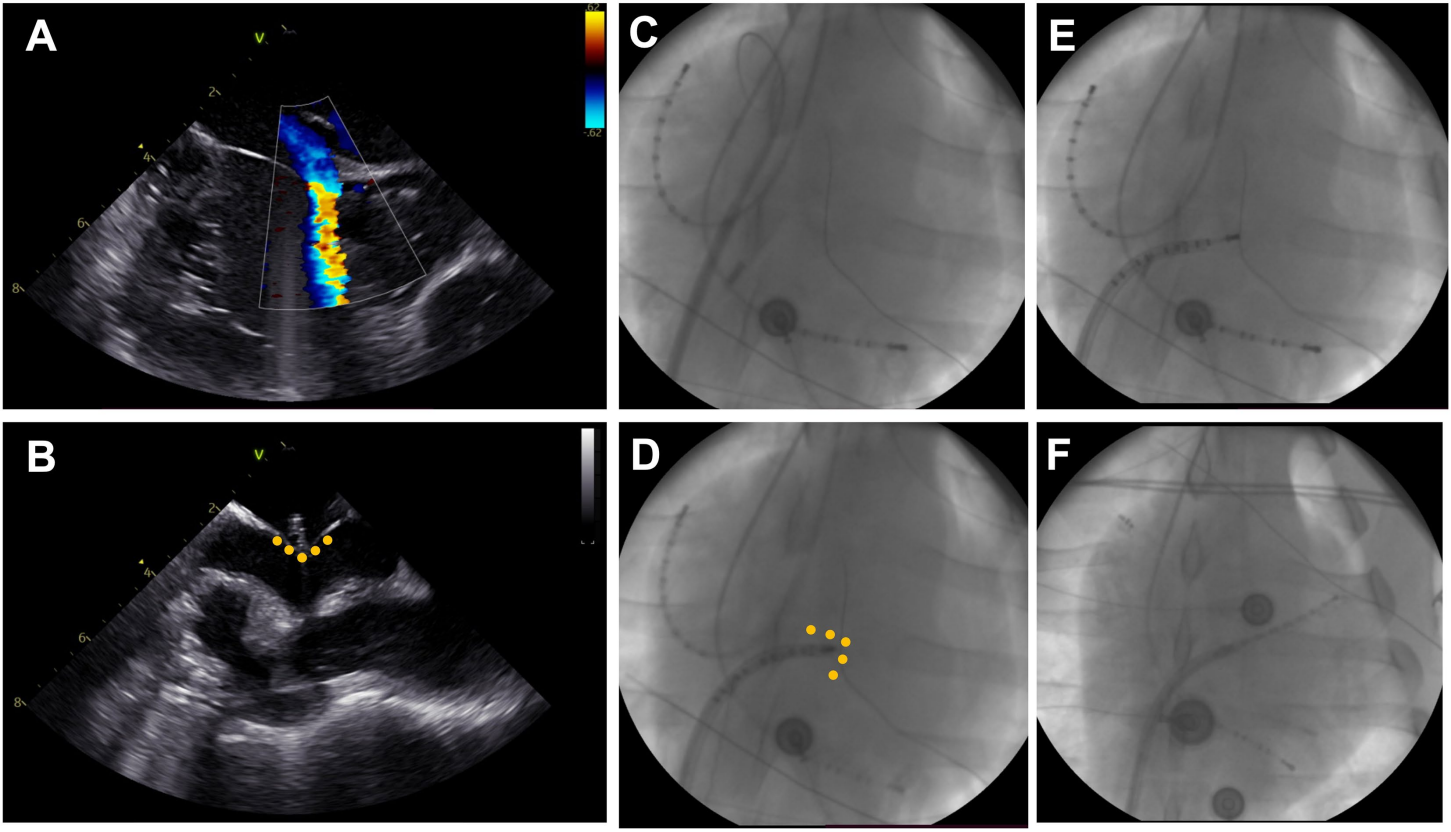
Transseptal puncture. **(A)** Visualization of a patent foramen ovale with right-to-left shunt on intracardiac echocardiography (ICE). **(B)** ICE-Visualization of blunt transseptal puncture through fossa ovalis showing tenting (yellow dots) and narrow left atrium. **(C)** Fluoroscopy image in anterior–posterior projection with decapolar catheter in high right atrium (left top) and coronary sinus (bottom right). The steerable sheath is advanced into the superior vena cava over the wire. **(D)** The sheath is retracted to the fossa ovalis and a diagnostic catheter is advanced. **(E)** Blunt passage of the fossa ovalis using the diagnostic catheter. **(F)** Positioning of the diagnostic catheter in the left atrial appendage and advancement of the steerable sheath into the left atrium.

Under fluoroscopic guidance (strict anterior–posterior projection) an intracardiac echocardiography (ICE) probe (ACUSON AcuNav™, Siemens, Muenchen, Germany) was advanced into the right atrium *via* the sheath in the left femoral vein to help visualize the interatrial septum (Figure 2). The short 8F sheath in the right femoral vein was exchanged for a long steerable sheath (Agilis NxT, 71 cm, medium curve, Abbott, Chicago, United States) and the wire advanced into the superior vena cava. Wire and dilator were removed, and a decapolar diagnostic catheter (Inquiry™, 6F, Abbott, Chicago, United States) introduced until the tip of the steerable sheath, which was deflected to 45°, turned to a 4 o’clock position and retracted into fossa ovalis. The diagnostic catheter was following advanced through the patent foramen ovale or pushed through the membranous part of the fossa ovalis, if possible. The transseptal passage was visualized in a subset of animals using ICE (Figures 2A–D). Successful transseptal puncture was verified with fluoroscopy (Figures 2E,F). The steerable sheath was advanced over the diagnostic catheter into the left atrium and flushed. In a subset of animals, selective pulmonary vein angiography, using a diagnostic coronary angiography catheter (Launcher 6\u00B0F, JL 3.5, 0.71″, Medtronic, Minneapolis, United States) and Visipaque 270 mg (GE Healthcare, Chicago, United States), was performed for illustration purposes. Finally, a multipolar mapping catheter (Advisor™ HD grid, Abbott, Chicago, United States) was introduced to perform the electroanatomic mapping.

### Electroanatomic mapping

All mapping procedures were performed using the EnSite Precision™ mapping system and the EnSite™ AutoMap function (Abbott, Chicago, United States). Electroanatomical maps were recorded in sinus rhythm using a high-density mapping catheter Advisor HD Grid Mapping Catheter, SE (Abbott Laboratories, Chicago, IL). The coronary sinus catheter was used as a reference electrode. For voltage maps, cut-offs of bipolar signals were set to 0.01–0.5 mV. Pulmonary vein locations were verified using fluoroscopy. We aimed to cannulate all branches previously described in the literature: one or more left superior branches, one or more right superior branches, inferior trunk with multiple branches (7).

### Pulmonary vein isolation

After left atrial electroanatomical mapping, the multipolar mapping catheter was exchanged for an ablation catheter (Tacticath™ Contact Force Ablation Catheter, Sensor Enabled™, Abbott, Chicago, United States). The pulmonary ostia were marked with a line on the electroanatomical mapping to guide ostial ablation. Ostial circumferential pulmonary vein isolation was performed by irrigated radiofrequency ablation with the following settings: 30 W, irrigation flow 25 mL/min, minimum inter-lesion distance 5 mm, 20–30 s application duration, target impedance drop of 15 Ohm, minimum impedance drop of 10 Ohm, or complete loss of local signal.

After encirclement of all previously mapped pulmonary veins, entrance- and exit-block were verified using the ablation catheter in at least five different pacing sites distal from every ablation line. Early reconnections were ruled out by re-assessing entrance- and exit-block after a 20 min waiting period (8). In a subset of animals, remapping after pulmonary vein isolation using the multipolar mapping catheter was performed for illustration purposes.

### *Post mortem* left atrial anatomical gross examination

At the end of the experiment, 10,000 IE heparin (Leo Pharma A/S, Lysaker, Norway) was injected intravenously, followed by a ten minute waiting period. The thorax was opened by mid-sternal thoracotomy, and the pleura was bluntly dissected to visualize the inferior vena cava. Animals were euthanised by cutting the inferior vena cava and draining all blood from the thorax. The heart was removed with attached lungs, trachea, esophagus and thymus, and the tissue was rinsed with physiological saline (0.9% NaCl). The pericardium, epicardium, lungs, pleura, trachea and esophagus were grossly examined, measured and photographed. A transverse cut was made to remove the ventricles. The left atrial appendage was opened at the anterior side to visualize the pulmonary ostia and veins, which were dissected open and excessive tissue was discarded.

**Figure 3:**
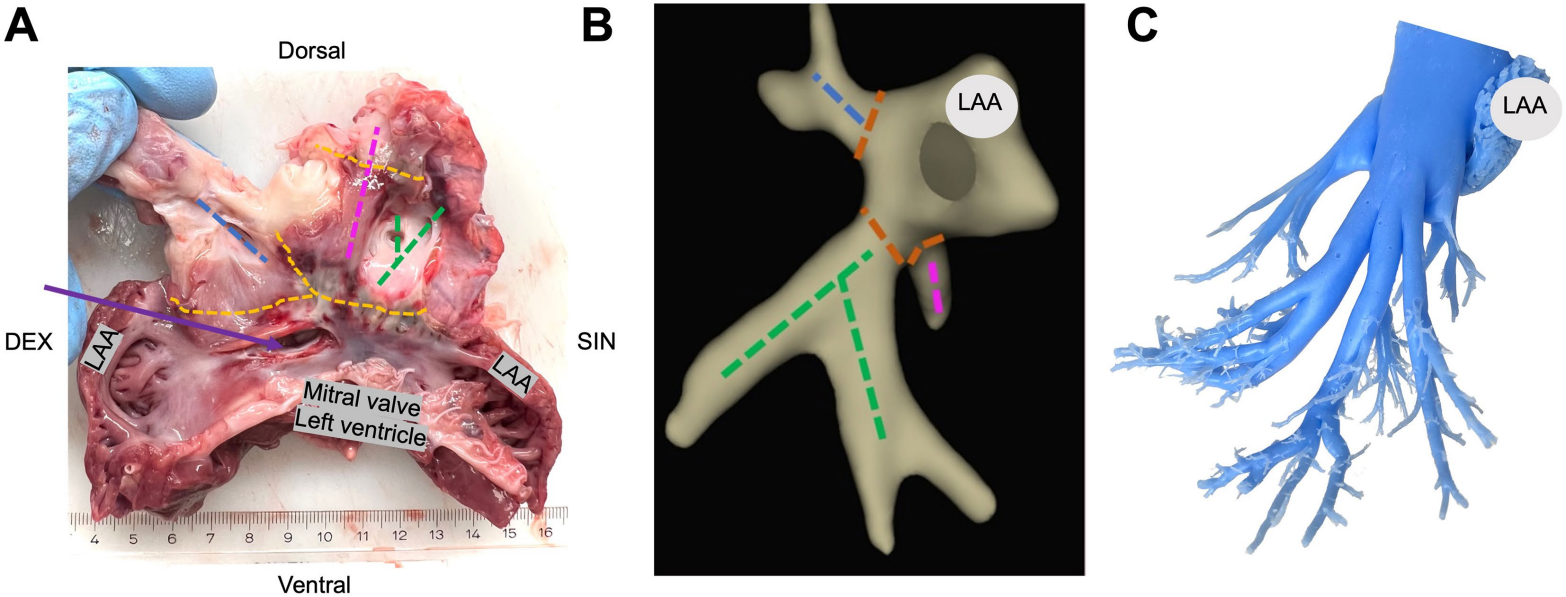
Gross examination and histological lesion assessment. **(A)** Gross examination of the excised left atrium cut through the left atrial appendage (LAA). A complete circumferential isolation (orange) around all pulmonary veins; the right superior pulmonary veins (blue), the left superior pulmonary veins (pink), and the common inferior pulmonary trunk (green). The transseptal puncture marked with purple arrow. **(B)** Electroanatomical map of the left atrium in anterior–posterior view with respective section site from panel A. **(C)** Silicone model of the left atrium showing pulmonary vein branches.

In a subset of animals, a silicone cast of the left atrium demonstrating pulmonary vein branching was created for illustrational purposes (Figure 3C; Supplementary Figure S2).

## Results

For this practical guide, we present procedural data derived from 11 consecutive Danish Landrace pigs (54.3 ± 2.5 kg and ~ 16 weeks old) undergoing left atrial electroanatomical mapping and pulmonary vein isolation by radiofrequency ablation.

### Groin access

Uncomplicated ultrasound-guided femoral vein access could be achieved in all animals (Figure 1). No bleeding or inadvertent arterial punctures occurred.

### Transseptal puncture

Successful and uneventful transseptal puncture and left atrial mapping could be performed in all 11 animals. In two animals, additional pulmonary vein angiography and in one animal, additional re-mapping after ablation was performed.

Due to the sagittal axis of the porcine heart in the thoracic cavity the ICE projections were rotated compared to normal projections in the human heart. Home view was found in a strict anterior angulation with the animal in a supine position. The interatrial septum was visualized with approximately 75–90° clockwise rotation of the probe. In 8/11 (73%) pigs, a patent foramen ovale was found and verified by leaflet separation and/or shunt in color Doppler mode (Figure 2).

**Figure 4:**
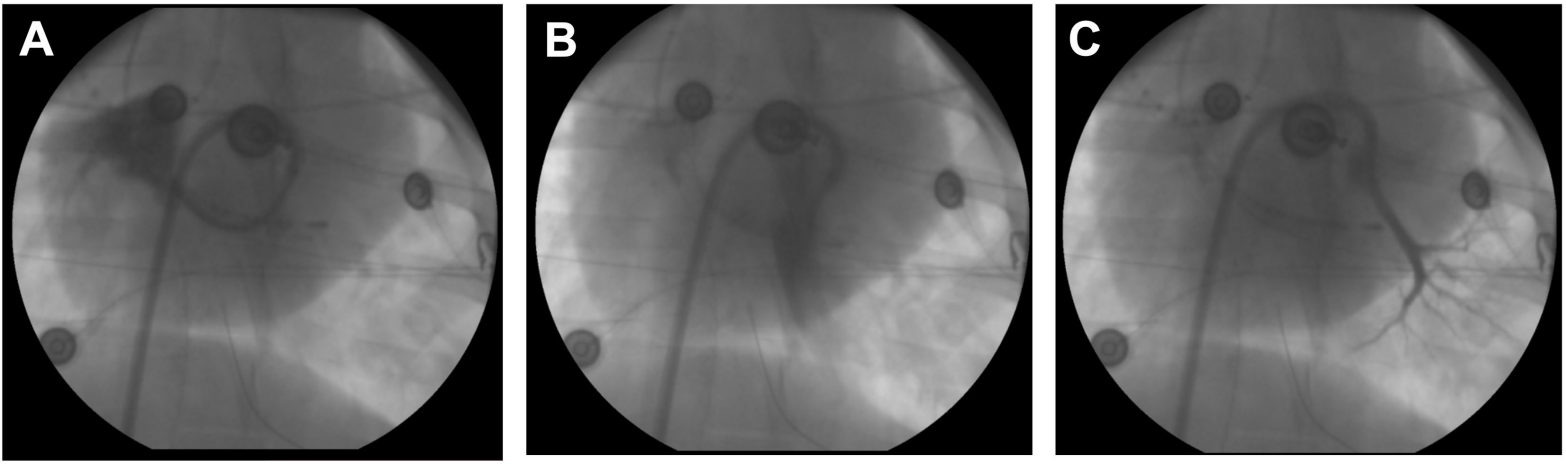
Pulmonary vein angiography. **(A)** Selective angiography of the right superior common ostium using a loop technique with steerable sheath and diagnostic catheter. **(B)** Angiography of the large inferior common trunk. **(C)** Angiography of the smaller left superior branch.

In animals with a patent foramen ovale, transseptal access was achieved by advancing the ablation catheter through the patent foramen ovale. In the remaining animals, the left atrium was accessed by a blunt puncture through the membranous part of the fossa ovalis. For illustrative purposes, selective pulmonary vein angiography was performed in a subset of animals displaying a small left superior pulmonary branch, a large inferior common pulmonary trunk, and a common trunk for the right inferior and right middle branches (Figure 4).

### Electroanatomical morphology of the left atrium

**Figure 5:**
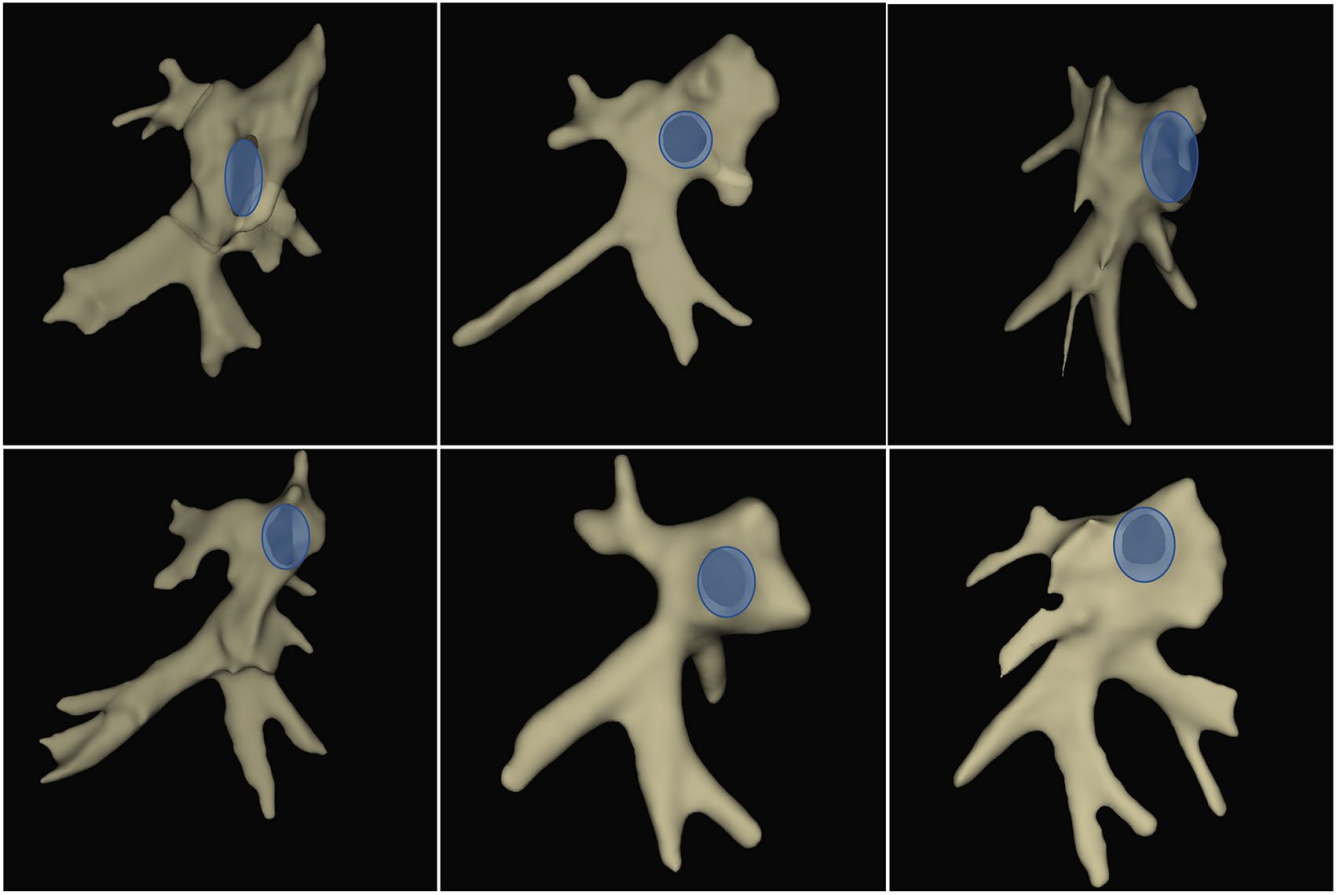
Pulmonary vein anatomy. All 3D-electroanatomic images are display in anterior–posterior projection facing the mitral valve (blue). All animals showed a common inferior trunk with a variable number of proximal branches, right middle and right superior branches arising from separate or one common ostium and a variable number of left middle and superior branches.

Electroanatomic mapping using a high-density mapping catheter identified different varieties of left atrial anatomy (Figure 5). The inferior common pulmonary trunk consisted of 2–4 branches. Superior to this trunk, 1–2 branches on each side could be mapped. Due to the narrow left atrial body and posterior position of the vein ostia, cannulation of the veins was challenging; however, it could occasionally be overcome by loop manoeuvres in the left atrium. A mean of 1,950 ± 612 points, including electroanatomical information of local bipolar EGMs (3D-location, bipolar EGM activation times and voltages), was used to create the maps of the left atria. Myocardial sleeves extended into all mapped pulmonary vein branches.

### Ablation data

**Figure 6:**
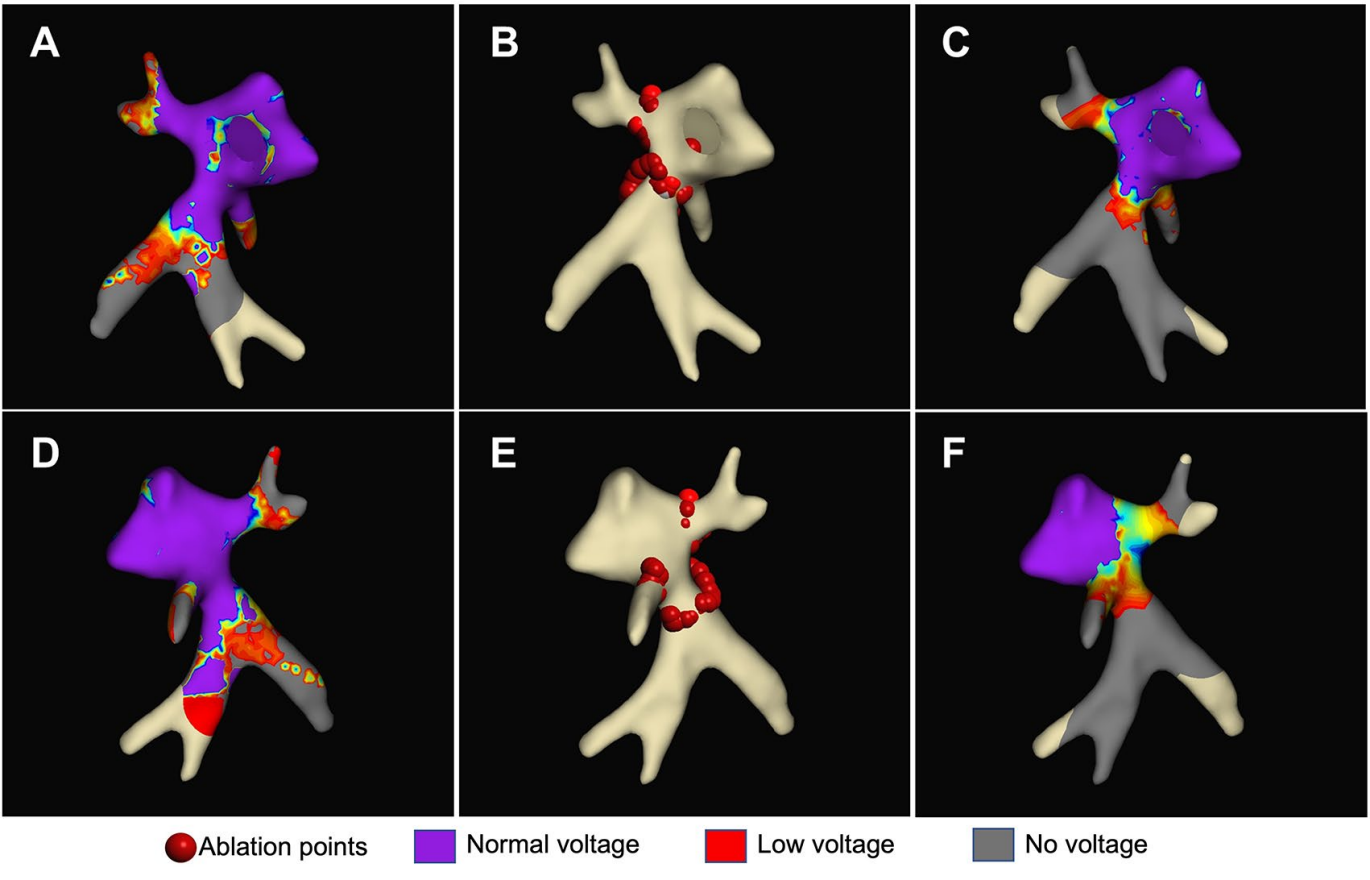
Pulmonary vein isolation. Left atrial maps in anterior–posterior **(A–C)** and posterior–anterior **(D–F)** projection. Voltage maps before **(A, D)** and after **(C, F)** selective circumferential pulmonary vein isolation points (red dots) (B + E: lesion set) with purple indication normal voltage (> 0.5 mV), blue to red indicating low voltage (0.1–0.5 mV) and grey indicating no voltage (<0.1 mV).

Successful electrical isolation of all mapped pulmonary veins was achieved in all animals (11/11, 100%) despite the flat LA anatomy and verified by entrance- and exit-block pacing. Figure 6 illustrates a high-density map of the left atrium before and after ablation, and the location of ablation points.

The portion inferior to the left superior pulmonary vein branch was problematic for ablation. In this location, we experienced phrenic nerve capture during high-output pacing in 3/11 (27.3%) animals (Supplementary Figure S1). When ablating more antral in this area, one animal went into recurrent ventricular fibrillation, which was successfully defibrillated. Further arrhythmias could be prevented by moving the ablation line more ostial. One of the animals showed transient phrenic nerve palsy after completion of radiofrequency ablation. No myocardial infarction, significant pericardial effusion or tamponade was observed.

### Macroscopic post-mortem lesion assessment

Inspection of the pulmonary veins, ablation lesions and transseptal puncture was performed in all animals. Dissection of the pulmonary veins was performed for illustrative purposes and gross examination. The gross examination revealed that all animals had a right superior pulmonary vein and a common inferior pulmonary trunk; nine animals had a left superior pulmonary vein, and three animals had a small pulmonary vein between the right superior pulmonary vein and the common inferior pulmonary trunk (Figure 3; Supplementary Figure S2). We found tearing of the fossa ovalis membrane in 3/11 (27%) animals where the transseptal puncture was performed.

## Discussion

To our knowledge, this is the first step-by-step practical guide on how to achieve a safe, complete, effective, and short-term durable catheter-based electrical pulmonary vein isolation in pigs.

Animal models are often used for mechanistic AF research since risk-factor-specific changes in humans are difficult to assess due to competing risk factors, high variability and limitations to access tissue samples (2). There are multiple large animal studies on the role of arterial hypertension, heart failure, inflammation or valvular heart disease on development and progression of AF (3). To accelerate the clinical implementation of findings in animal models, it is important to apply clinically relevant therapeutic interventions. In the case of pulmonary vein isolation, the use of lesion sets comparable to humans is essential. We demonstrated that although the anatomy of the left atrium is substantially different compared to humans, electrical isolation of the myocardial sleeves, which extended in all mapped pulmonary veins, can be safely achieved using the currently available electroanatomical mapping and ablation technologies with clinically approved equipment.

Previous studies have used pig models to test different innovative ablation technologies such as the laser-balloon, the low-intensity collimated ultrasound system, the diamond tip ablation catheter, impedance-guided ablations and the different pulsed field ablation catheter (9–16). All of these studies investigated single ablation lesions without the specific goal of achieving complete pulmonary vein isolation. Other earlier studies showed that establishing left atrial lesion sets in pigs can be challenging and complicated by high rates of problematic transseptal punctures, pericardial tamponades, perforations, haemorrhages and deaths in a large proportion of animals (17). This step-by-step practical guide will help other research groups in developing pulmonary vein isolation in large animal models, reduce the need for pilot experiments and reduce procedure-related complications in line with the 3R principle.

### Transseptal puncture

When positioned in a supine position, the left atrium appears compressed on electroanatomic maps and ICE in the pig. This might explain the high rates of tamponades in earlier studies when transseptal puncture was performed with needles as done in humans (17). In our study, we used a patent foramen ovale, which was present in the majority of animals, to advance catheters into the left atrium. We report a higher prevalence of patent foramen ovale than reported previously in other studies, which could be due to the relatively young age of the animals in our study (18). In case there was no patent foramen ovale, we positioned the steerable sheath in the membranous part of the fossa ovalis, and passed it with a diagnostic catheter with only very little force. This technique achieved quick, safe, and reproducible transseptal puncture in all animals. Post-mortem, we observed some tears in the fossa ovalis membrane in those animals where a transseptal puncture was required. To achieve electrical isolation of all pulmonary veins, the sheath and catheter movements inside the flat atrium often needed additional rigorous movements than typically performed in human pulmonary vein isolation procedures, which may have contributed to the damage of the thin-walled interatrial septum (19). The usage of ICE catheters is feasible in pigs and helped to establish the transseptal puncture during fluoroscopy by visualizing the pig heart anatomy. However, ICE it is not required to obtain reproducible results with fluoroscopy-guided transseptal puncture.

### Mapping and ablation

There are important pig-specific anatomical considerations for left atrial mapping. The pig’s left atrial appendage takes up a larger proportion of the atrium, the atrial body is smaller, and up to eight specific pulmonary vein anatomies have been described. Usually, the right cranial and middle lung lobe drain into a right superior common ostium, and the inferior lobes drain into a common inferior trunk that can also drain the left superior branch. When considering the limited manoeuvrability within the small atrial body, larger sheaths and single-shot devices such as cryo-balloons or pulsed-field ablation basket catheters may be useful to ablate the common inferior trunk, while the smaller pulmonary vein branches are likely difficult to reach. We could overcome these limitations using bidirectional steerable sheaths combined with multipolar mapping catheters and point-by-point ablation catheters with small curves. We also found the best pulmonary vein isolation results when performing encirclement at the left atrial pulmonary vein junction. This has implications for post-procedural pulmonary vein tissue sampling for lesion assessment in pigs; thus, the heart should be removed attached to the lungs, trachea, esophagus and thymus to avoid ostial excision of the pulmonary trunks.

## Limitations

The electroanatomical mapping and ablation procedures in this study were performed by three electrophysiologists experienced in performing pulmonary vein isolation in humans. A longer learning curve may be required for operators not skilled in left atrial electrophysiological studies. Only one of the many available mapping systems was used, so results might not be transferrable to other mapping systems and ablation techniques. According to current expert consensus for AF ablation in humans, the traditional 20 min waiting period was used to account for early pulmonary vein reconnections (8). For the assessment of true durability, re-mapping or histological examinations after three months would be required, which was not the scope of this study.

## Conclusion

Following this step-by-step approach, fluoroscopic- and intracardiac ultrasound- guided transseptal puncture, high-density electroanatomical mapping of the left atrium and all pulmonary veins, as well as complete electrical pulmonary vein isolation can be achieved reproducibly and safely in pigs.

## Data availability statement

The original contributions presented in the study are included in the article/Supplementary material, further inquiries can be directed to the corresponding author.

## Ethics statement

The animal study was reviewed and approved by the experimental protocol was approved by the Regional Animal Welfare Inspectorate (Miljø- og Fødevareministeriet: license #2018-15-0201-01608) and conforms with the guidelines from Directive 2010/63/EU of the European Parliament on the protection of laboratory animals and conforms with the Guide for the Care and Use of Laboratory Animals published by the US National Institute of Health (NIH publication no. 85-23, revised 1996).

## Author contributions

All authors listed have made a substantial, direct, and intellectual contribution to the work and approved it for publication.

## Funding

This work was funded by NNF Young Investigator Awards 2021 (NNF21OC0066480) to DL; Independent Research Fund Denmark (102900011B) to AS; and NNF Tandem Programme (NNF18OC0031634) to TJ.

## Conflict of interest

The authors declare that the research was conducted in the absence of any commercial or financial relationships that could be construed as a potential conflict of interest.

## Publisher’s note

All claims expressed in this article are solely those of the authors and do not necessarily represent those of their affiliated organizations, or those of the publisher, the editors and the reviewers. Any product that may be evaluated in this article, or claim that may be made by its manufacturer, is not guaranteed or endorsed by the publisher.
