# Supplementary figures and images for: 3D-electroanatomical mapping of the left atrium and catheter-based pulmonary vein isolation in pigs: A practical guide
Source: Front Cardiovasc Med. 2023 Mar 9;10:1139364. doi: 10.3389/fcvm.2023.1139364 (PMC10033609; doi:10.3389/fcvm.2023.1139364)

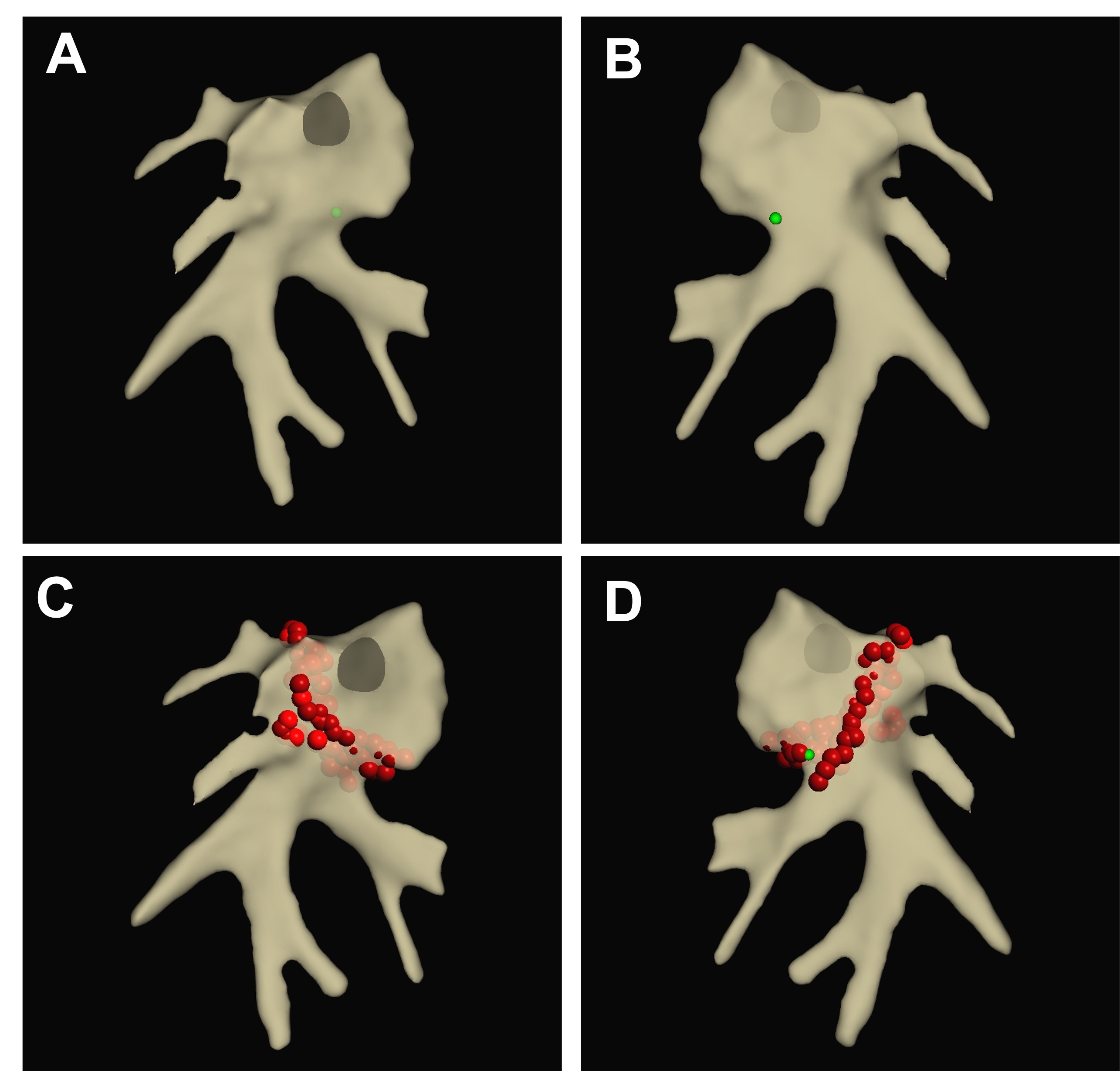

Supplement: Supplementary file 1 [file Image_1.jpeg]

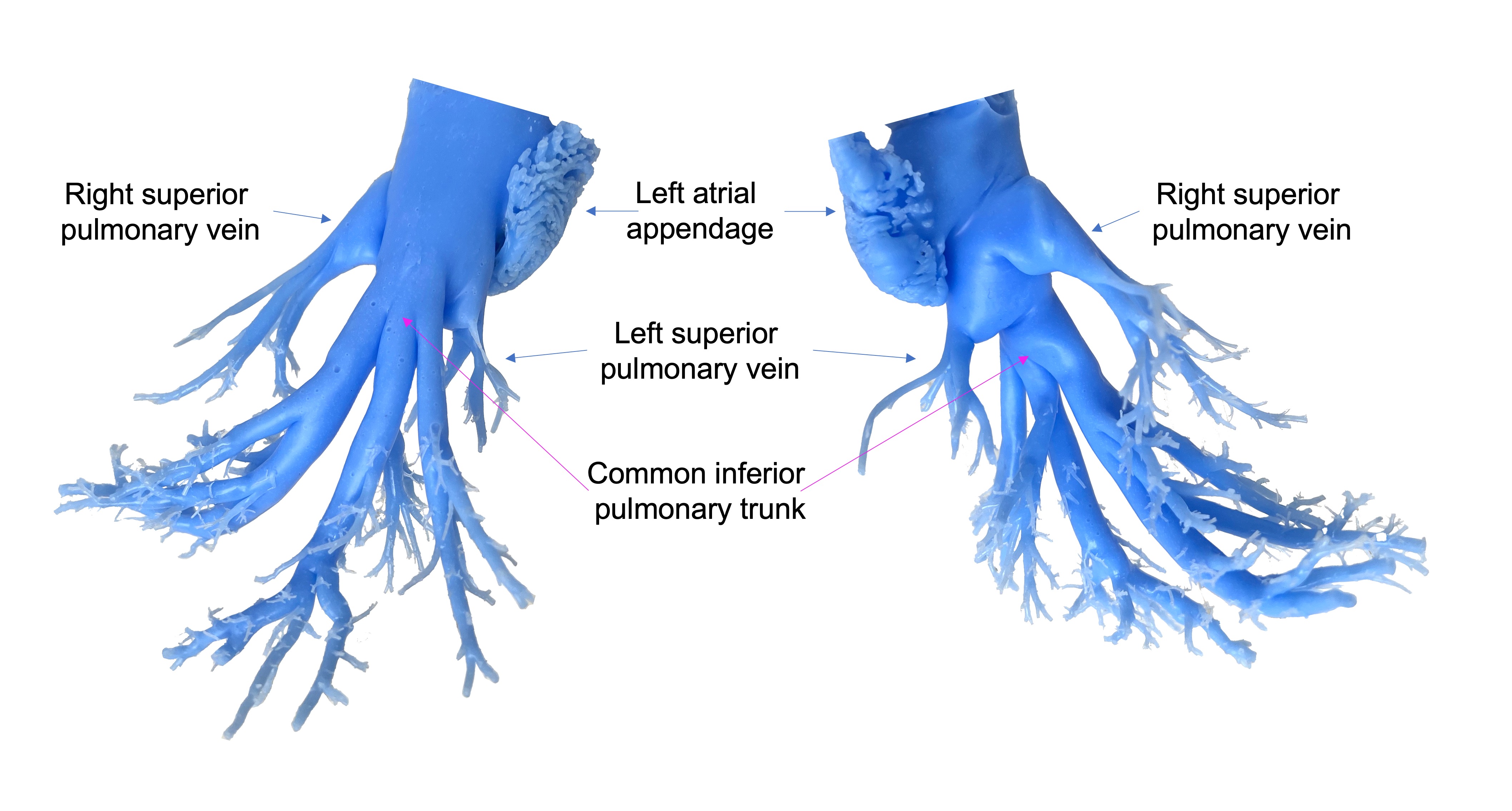

Supplement: Supplementary file 2 [file Image_2.jpeg]
