# Supplementary material for: 3D-electroanatomical mapping of the left atrium and catheter-based pulmonary vein isolation in pigs: A practical guide
Source: Front Cardiovasc Med. 2023 Mar 9;10:1139364. doi: 10.3389/fcvm.2023.1139364 (PMC10033609; doi:10.3389/fcvm.2023.1139364)
Supplement: Supplementary file 3 [file Table_1.DOCX]

3D-electroanatomical mapping of the left atrium and catheter-based pulmonary vein isolation in pigs: A practical guide.

Manuscript ID: 1139364

Dear members of the editorial team,

Please find the attached revision of our manuscript for publication in Frontiers in Cardiovascular Medicine. A detailed list of changes after the reviewer’s comments and all changes are highlighted in red in the revised version of the manuscript.

All authors have read and approved the revised version of the manuscript. The manuscript has not been published online, nor is it considered for publication elsewhere.

We hope the Frontiers Journal and its reviewers accept the revised version of the manuscript.

Independent Review Report, Reviewer 1

The present Manuscript by Hertel and colleagues proposes a standardised workflow for 3D-electroanatomical mapping guided pulmonary vein isolation in pigs. The authors concluded that transseptal puncture, high-density electroanatomical mapping of all pulmonary veins and complete electrical pulmonary vein isolation can be achieved reproducibly and safely in pigs when using current technologies and a step-by-step approach. The authors are congratulated on this work.

Minor questions:

- What was the purpose of dissection after PVI? Were histological tests performed?

We thank the reviewer for the important questions. Dissection after PVI was performed to inspect the ablation lesions around the pulmonary ostia macroscopically, to examine the transseptal lesions and for illustrative purposes. Tissue has been preserved for later histological assessment of the acute ablation lesions.

We have clarified this in the revised manuscript adding the following:

Results, macroscopic post-mortem lesion assessment ll. 246-248:

‘Inspection of the pulmonary veins, ablation lesions and transseptal puncture	was performed in all animals. Dissection of the pulmonary veins was performed	for illustrative purposes and gross examination.’

- Is it really necessary to use ICE for transseptal puncture in pigs? Have the authors attempted fluroscopic TSP in some cases?

We thank the reviewer for the questions. The ICE catheters were used to help guide the transseptal puncture during fluoroscopy and helped understand the porcine anatomy and explore the prevalence of persistent foramen ovale. However, using ICE for the TSP in pigs is not necessary; thus, fluoroscopic guidance of TSP was used in all animals.

We have clarified this with cursive in the revised version of the manuscript the following:

Methods, transseptal puncture ll. 130-133

‘Under fluoroscopic guidance (strict anterior-posterior projection) an intracardiac	echocardiography (ICE) probe (ACUSON AcuNav™, Siemens, Muenchen,	Germany) was advanced into the right atrium via the sheath in the left femoral	vein to *help* visualise the interatrial septum’

Discussion, transseptal puncture ll. 299-302;

‘The usage of ICE catheters is feasible in pigs and helped to establish the	transseptal puncture during fluoroscopy by visualizing the pig heart anatomy.	However, ICE it is not required to obtain reproducible results with fluoroscopy-	guided transseptal puncture.’

- How were the PV ostia marked before ablation?

We thank the reviewer for this important comment. The pulmonary vein ostia were marked with a guiding line on the electroanatomical map to direct each ablation.

We have clarified this by adding the following to the revised version:

Methods, pulmonary vein isolation ll. 164-165.

‘The pulmonary ostia were marked with a line on the electroanatomical mapping	to guide ostial ablation.’

- Why was selective angiography performed only in one animal?

We thank the reviewer for this question. The selective angiography of the pulmonary veins was performed in a subset of animals, and the angiography from one animal was selected for this manuscript for illustrative purposes.

We have clarified this by adding the following in cursive;

Results, Transseptal puncture ll. 215-218

*‘For illustrative purposes,* selective pulmonary vein angiography was performed	in *a subset of animals displaying* a small left superior pulmonary branch, a large	inferior common pulmonary trunk, and a common trunk for the right inferior and	right middle branches (Figure 3).’

Check List

a. Is the quality of the figures and tables satisfactory?

Yes
b. Does the reference list cover the relevant literature adequately and in an unbiased manner?

Yes
c. Are the statistical methods valid and correctly applied? (e.g. sample size, choice of test)

Yes
d. Is a statistician required to evaluate this study?

No
e. Are the methods sufficiently documented to allow replication studies?

Yes
f. Are limits of detection or quantification adequately described?

Yes
QUALITY ASSESSMENT:

Rigor
3
Quality of the writing

3
Overall quality of the content

3
Interest to a general audience

2

Independent Review Report, Reviewer 3

Major comments:

This is an excellent report on a pig model for EP procedures. The authors propose a standardized workflow for high-resolution 3D mapping in pigs with current mapping technology and RF contact force sensing catheters.

The manuscript is well written, the figures and the presentation of the data is appropriate and the discussion is interesting and fair. Limitations might be the low number of pigs studied with 3 investigators and their individual learning curve. Some revisions and update of the references are recommended (see below).

We thank the reviewer for this comment. For the refinement of the transseptal puncture approach and electroanatomical mapping, three experienced electrophysiologists were involved during all experiments, and their combined inputs were included for safe and reproducible step-by-step development of the approach. As the electrophysiologists are experienced in human pulmonary vein isolation and familiar with using the Ensite system and performing RF-ablations, their learning curves were fast, and fewer animals were needed to establish the protocol. However, if the researchers were inexperienced, a longer learning curve and a larger population could be expected.

Minor comments:

- Abstract: As ICE guidance for PVI is not established in all labs, the reviewer suggests to add “Intracardiac ultrasound-guided” to the beginning of the first sentence of the conclusion of the abstract.

We thank the reviewer for the comment, we have clarified in the abstract and in the conclusion as well.

See abstract, under conclusion ll. 58-59 in cursive:

*‘Intracardiac ultrasound and fluoroscopic-guided* transseptal puncture, high-	density electroanatomical mapping of all pulmonary veins and complete	electrical pulmonary vein isolation can be achieved reproducibly and safely in	pigs when using current technologies and a step-by-step approach.’

Discussion, transseptal puncture ll. 299-302;

‘The usage of ICE catheters is feasible in pigs and helped to establish the	transseptal puncture during fluoroscopy by visualizing the pig heart anatomy.	However, ICE it is not required to obtain reproducible results with fluoroscopy-	guided transseptal puncture.’

And in the conclusion, l. 334-337:

‘Following this step-by-step approach, fluoroscopic- and intracardiac ultrasound-	guided transseptal puncture, high-density electroanatomical mapping of the left	atrium and all pulmonary veins, as well as complete electrical pulmonary vein	isolation can be achieved reproducibly and safely in pigs.’

- Contribution to the field: Is there a typing error in this sentence with a “6” included?

“as presented in our manuscript, supports the principle within the 3Rs (replacement, reduction, refinement)6 framework for performing more humane animal research”

We thank the reviewer for this observation. We have moved the misplaced “6”-reference to the end of the sentence. See l. 76.

Discussion: “The usage of ICE catheters helped guide during the transseptal puncture and understand…” – I would suggest to include “is feasible in pigs and helped…”

We thank the reviewer for this suggestion, and we have changed the following in cursive in the discussion, ll. 299-302:

‘The usage of ICE catheters is feasible in pigs and helped to establish the	transseptal puncture during fluoroscopy by visualizing the pig heart anatomy.	However, ICE it is not required to obtain reproducible results with fluoroscopy-	guided transseptal puncture.’

Conclusion: please consider “intracardiac ultrasound guided transseptal puncture” to add in the first sentence of the conclusion (see abstract comment)

We thank the reviewer for this comment, and we have added to the conclusion (see abstract comment).

References:

You might add recent publication to the manuscripts focusing on new technology and PVI evaluated in pig models and compare those models to your system in the discussion section e.g.

• Lattice Tip: DOI: 10.1007/s10840-022-01414-7.

• Groen et al contact measurement in PFA: doi: 10.1161/CIRCEP.121.010835.

• Time Course of Irreversible Electroporation Lesion doi: 10.1161/CIRCEP.121.010661.

• Local impedence guided ablation: doi: 10.1111/jce.15135.

We thank the reviewer for recommending the four publications, we have added these to the discussion.

See discussion ll. 272-275:

‘Previous studies have used pig models to test different innovative ablation	technologies such as the laser-balloon, the low-intensity collimated ultrasound	system, the diamond tip ablation catheter, impedance-guided ablations and the	different pulsed field ablation catheter.^9–16^’

Check List

a. Is the quality of the figures and tables satisfactory?

Yes

b. Does the reference list cover the relevant literature adequately and in an unbiased manner?

Yes

c. Are the statistical methods valid and correctly applied? (e.g. sample size, choice of test)

Yes

d. Is a statistician required to evaluate this study?

No

e. Are the methods sufficiently documented to allow replication studies?

Yes

f. Are limits of detection or quantification adequately described?

Yes

QUALITY ASSESSMENT:

Rigor

4

Quality of the writing

5

Overall quality of the content

5

Interest to a general audience

4
